# Discovery of plasma biomarkers related to blood-brain barrier dysregulation in Alzheimer’s disease

**DOI:** 10.3389/fbinf.2024.1463001

**Published:** 2024-10-04

**Authors:** Yuet Ruh Dan, Keng-Hwee Chiam

**Affiliations:** ^1^ School of Biological Sciences, Nanyang Technological University, Singapore, Singapore; ^2^ Bioinformatics Institute, A*STAR, Singapore, Singapore

**Keywords:** alzheimer’s disease, plasma biomarkers, blood-brain barrier, proteomics analysis, single-cell transcriptomics

## Abstract

**Introduction:**

Blood-based biomarkers are quantitative, non-invasive diagnostic tools. This study aimed to identify candidate biomarkers for Alzheimer’s disease (AD) using publicly available omics datasets, using the hypothesis that with blood-brain barrier dysfunction in AD, brain-synthesized proteins can leak into plasma for detection.

**Methods:**

Differential abundance results of plasma and brain proteomic datasets were integrated to obtain a list of potential biomarkers. Biological validity was investigated with intercellular communication and gene regulatory analyses on brain single-cell transcriptomics data.

**Results:**

Five proteins (APOD, B2M, CFH, CLU, and C3) fit biomarker criteria. 4 corresponding transcripts (APOD, B2M, CLU, and C3) were overexpressed in AD astrocytes, mediated by AD-related dysregulations in transcription factors regulating neuroinflammation. Additionally, CLU specifically induced downstream expression of neuronal death genes.

**Discussion:**

In conclusion, a 5-protein panel is shown to effectively identify AD patients, with evidence of disease specificity and biological validity. Future research should investigate the mechanism of protein leakage through the blood-brain barrier.

## 1 Introduction

First discovered in 1906, Alzheimer’s disease (AD) is now one of the most common neurodegenerative diseases. Clinically, AD is characterized by amnestic cognitive impairments, and neuropathologically, patients possess a signature of extracellularly accumulated β-amyloid (Aβ) plaques and intracellular neurofibrillary tangles. AD accounts for up to 80% of all dementias (DeTure and Dickson, 2019) and costs US$1 trillion yearly (Livingston et al., 2020).

Diagnostic criteria for AD are subject to significant debate (Dubois et al., 2021). Amyloid positivity is the main employed quantitative criterion, assessed through brain positron emission tomography (PET) imaging or soluble Aβ levels in cerebrospinal fluid (CSF). However, both are undesirable for large-scale applications, as PET carries a significant financial cost, and the collection of CSF entails an invasive lumbar puncture. Furthermore, pure amyloid positivity is not sufficient for an AD diagnosis, due to complex comorbidities and the presence of amyloid deposits in cognitively normal patients (Dubois et al., 2018). This renders neurocognitive testing as the bottom line in AD diagnosis, a time-consuming and insensitive process (Carson et al., 2018; Spering et al., 2012). Evidently, current methods of diagnosis are subjective, cost-inefficient, and difficult to administer. Novel diagnostic tools are thus needed.

Since samples can be obtained cheaply and easily, blood-based biomarkers (BBMs) are increasingly touted as the ideal diagnostic tool. Studies have evaluated the use of BBMs in determining amyloid positivity, disease diagnosis, as well as risk of disease development, leveraging on the development of high-throughput omics to examine the plasma transcriptome, proteome, and metabolome. For example, with a plasma panel of five proteins, Burnham et al. (Burnham et al., 2014) predicted neocortical amyloid positivity with sensitivity and specificity values of above 75%. However, though many studies have since employed similar methodologies and published promising results, progress is limited by significant inter-study heterogeneity and poor reproducibility (O’Bryant et al., 2017). A more theory-driven approach may thus be warranted to increase validity of identified BBMs across samples and assay methods.

In this study, dysregulation of the blood-brain barrier (BBB) in AD was investigated as a potential mechanism for plasma protein dysregulation. Comprising a continuous monolayer of endothelial cells, transport of macromolecules across the BBB is typically greatly inhibited by junctional complexes such as tight junctions and adherens junctions (Knox et al., 2022) which affect paracellular transport, as well as low endogenous levels of transendothelial transport (Sweeney et al., 2018). However, breakdown of this barrier has been well-documented in AD. Enhanced permeability of the BBB to fluorescent markers has been shown in AD mouse models (Liu et al., 2020), together with decreased tight junction expression and evidence of morphological vessel damage (Islas-Hernandez and Garcia-Delatorre, 2020). In humans, magnetic resonance imaging has also depicted increased permeability of peripheral contrast agents into brain tissues (Sweeney et al., 2018) and increased microhemorrhages in the cortices of AD subjects (van de Haar et al., 2016) suggesting increased tissue extravasation of red blood cells. As such, we hypothesised that as the integrity of the BBB weakens with AD, proteins which are initially sequestered in the brain will be able to leak out into the peripheral circulation, resulting in some proteins being upregulated in AD plasma compared to healthy control plasma. In particular, given that AD is a largely protopathic disease (Johnson et al., 2022), proteins upregulated in the brain as a consequence of AD-specific processes may be able to be transported into the plasma, serving as BBMs that could diagnose the presence or absence of AD.

**FIGURE 1:**
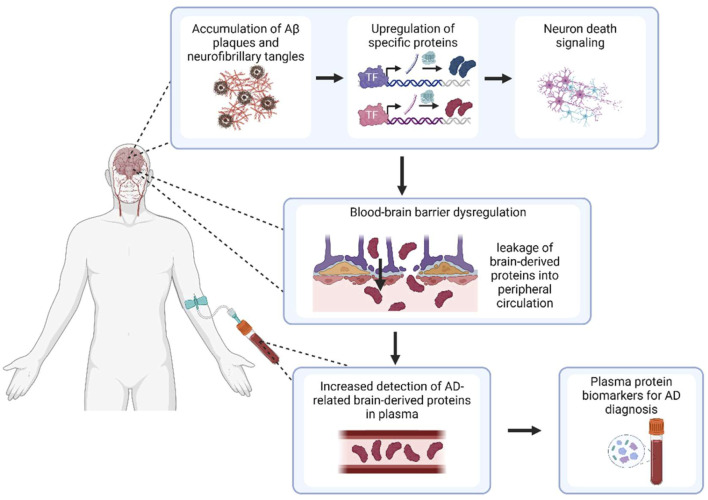
Overview of study hypothesis for detection of BBMs for AD. Created with BioRender.com

Based on this theoretical underpinning, we aimed to identify dysregulated plasma proteins that were upregulated in the AD brain and transported through the faulty BBB, thus providing future clinical and experimental investigations with reliable, data-driven candidates for AD BBM validation (Figure 1). Overall, five proteins were identified: apolipoprotein D (APOD), beta-2 microglobulin (B2M), complement 3 (C3), clusterin (CLU), and complement factor H (CFH). These proteins were investigated as a 5-protein BBM panel, and were assessed for effectiveness, specificity and validity as an AD diagnostic panel.

## 2 Methods

**FIGURE 2:**
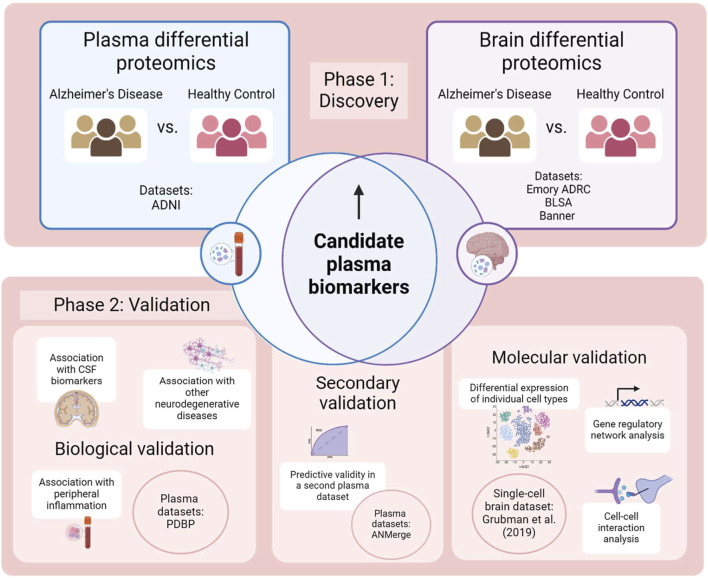
Methodology employed by this study. Created with BioRender.com.

The overall methodological framework of this study is illustrated in Figure 2. Briefly, in the discovery phase, differential abundance analysis was performed separately on plasma proteomics data and brain proteomics data, and a list of proteins that were significantly overabundant in AD plasma and brains were curated as potential plasma biomarkers. Validation was assessed in three prongs: biological validity of the plasma panel was confirmed by investigating its association with markers of AD pathology, peripheral inflammation and other neurodegenerative diseases, while predictive validity of the panel was ascertained in a secondary unseen plasma dataset. Finally, molecular validation was assessed by studying upstream mechanisms of protein dysregulation in the brain, to verify that the changes were AD-specific.

### 2.1 Study cohorts

Three plasma proteomics cohorts, three brain proteomics cohorts and one single-cell RNA (scRNA) cohort consisting of AD and healthy control (HC) samples were analysed in this study. All datasets used are publicly available. Brain proteomics data was generated from postmortem brain tissue collected through the Emory Alzheimers’ Disease Research Center Brain Bank, The National Institute on Aging’s Baltimore Longitudinal Study of Aging and the Sun Health Research Institute Brain and Body Donation Program of Sun City, Arizona. All brain proteomic cohorts and the ANMerge plasma proteomic cohort were accessed from the AD Knowledge Portal hosted on Synapse (https://adknowledgeportal.org) and downloaded with their corresponding metadata. The scRNA dataset and metadata was downloaded from its website (http://adsn.ddnetbio.com/) (Grubman et al., 2019). The remaining AD plasma proteomics dataset used was obtained from the Alzheimer’s Disease Neuroimaging Initiative (ADNI) database, while the Parkinson’s Disease Biomarker Program (PDBP) plasma proteomics data was accessed and downloaded as a Supplementary Table from Posavi et al. (2019). The datasets are listed in Table 1.

**TABLE 1:** Datasets used.

Study	Samples	Tissue	Assay type	Accessed from
-^a^	Healthy control, Alzheimer’s disease	Entorhinal cortex	Single-cell transcriptomics	Grubman et al. (2019)
ANMerge	Healthy control, Alzheimer’s disease	Blood plasma	Proteomics	Available on Synapse: syn22252881
Alzheimer’s Disease Neuroimaging Initiative (ADNI)	Healthy control, Alzheimer’s disease	Blood plasma	Proteomics	Petersen et al. (2010)
National Institute of Neurological Disorders and Stroke Parkinson’s Disease Biomarker Program	Healthy control, Amyotrophic lateral sclerosis, Parkinson’s disease	Blood plasma	Proteomics	Posavi et al. (2019)
Banner Sun Health Research Institute Study	Healthy control, Alzheimer’s disease	Dorsolateral prefrontal cortex	Proteomics	Available on Synapse: syn7170616
Baltimore Longitudinal Study on Aging (BLSA) Study	Healthy control, Alzheimer’s disease	Dorsolateral prefrontal cortex	Proteomics	Available on Synapse: syn3606086
Emory Alzheimer’s Disease Research Center (Emory ADRC) Brain Bank Study	Healthy control, Alzheimer’s disease	Dorsolateral prefrontal cortex	Proteomics	Available on Synapse: syn3218563

^a^ Not part of a program.

### 2.2 Data preprocessing

Prior to downloading, the ADNI plasma proteomics dataset had undergone quality control performed by the Consortium, namely, removal of analytes with more than 10% missing data, transformation of non-normal protein data, imputation of missing values, and removal of outliers. Demographics are presented in Table 2. Subsequently, data was filtered for protein abundance measurements performed during baseline visits, and two samples were excluded due to discrepancies in the recorded diagnosis. The variance of each protein abundance contributed by age was calculated using linear regression, and it was found that seven proteins had more than 10% of their variances explained by age. The effect of age was thus corrected for with linear regression, and this age-adjusted dataset consisting of 146 proteins and 564 samples was used for downstream analysis.

**TABLE 2:** Demographics of plasma proteomics data.

	ADNI plasma proteomics		ANMerge plasma proteomics		PD-BP plasma proteomics		
	AD	HC	AD	HC	PD	ALS	HC
n	111	453	430	269	215	59	102
Age^a^	74.73 (8.07)	74.77 (7.21)	77.73 (6.35)^b^	75.25 (5.36)	66.7 (8.55)	61.9 (10.5)	66.1 (10.5)
Gender (Male/Female)	64/47	285/168	148/383	125/144	114/101	33/26	51/51

^a^ Data is presented as mean (standard deviation).

^b^ n = 399.

The ANMerge plasma proteomics dataset was log transformed and corrected for batch processing effects. No further preprocessing was conducted, and the final dataset consisted of 1,001 unique proteins and 699 samples. PDBP plasma proteomics had similarly been log-transformed prior to downloading, and no significant batch effects were observed thus no batch adjustment was performed. A total of 1,305 proteins were measured in 376 samples. Demographics of both cohorts are presented in Table 2.

Individually, brain proteomic datasets from the Emory ADRC study, the BLSA study, and the Banner study were filtered for potential analyte contaminants, analytes which were only identified by a modification site, analytes part of a decoy database, analytes with non-gene symbol names. Duplicate measurements were averaged into a single column. Summary statistics are presented in Table 3. Since all datasets utilized the same label-free LC-MS/MS methodology with MaxQuant quantification, normalized intensity data columns of each dataset were subsequently merged for meta-analysis. Dataset-specific correction was performed with the ComBat function in the R package sva (Johnson et al., 2007), and the dataset was filtered for proteins detected in less than 10% of samples. Imputation of missing values was subsequently performed using the R package missForest (Stekhoven and Bühlmann, 2012), a random forest classification-based imputation algorithm. The final dataset used for differential protein analysis consisted of 2,955 proteins and 250 samples.

**TABLE 3:** Demographics of brain proteomics data.

	Emory ADRC		Banner		BLSA	
	AD	HC	AD	HC	AD	HC
n	8	8	100	101	20	13
Age^a^	64.50 (5.83)	65.75 (6.54)	83.25 (6.18)	85.31 (5.22)	84.75 (7.25)	80.15 (9.27)
Gender (Male/Female)	4/4	5/3	58/42	56/45	10/10	10/3
Brain region	Middle frontal gyrus		Middle frontal gyrus		Middle frontal gyrus	

^a^ Data is presented as mean (standard deviation). Where age was coded as ‘≥90’, the numerical value of 90 was taken for calculation of summary statistics.

### 2.3 Differential protein abundance analysis

Differential protein abundance analysis was performed on plasma proteomics datasets as well as the combined brain proteomic dataset using the Wilcoxon rank-sum test, with Benjamin-Hochberg correction. An adjusted cut-off *p*-value of 0.05 was used.

Correlational analysis of the differentially abundant proteins in the brain proteomic dataset was further performed against fibrinogen. Fibrinogen is only synthesized in the periphery and not the brain, thus fibrinogen levels in the brain are a well-established marker of BBB permeability. An adjusted cut-off *p*-value of 0.05 was used.

### 2.4 Validation of BBM panel

Random forest models using the R package randomForest (Liaw and Wiener, 2002) were built using the candidate BBMs as features, using default parameters. The R package caret was used to subset plasma proteomic data into training and testing data at a split of 0.8, and subsequently the R package pROC (Robin et al., 2011) was used to test the random forest models on diagnosis prediction. Receiver operator curves (ROC) and the corresponding area under the ROCs (AUROC) were obtained with the original ADNI plasma proteomics data, as well as with ANMerge plasma proteomics data, which served as a separate validation dataset.

### 2.5 CSF biomarker-corrected plasma BBM analysis

340 samples from the ADNI plasma proteomics dataset were able to be matched to CSF samples. Thus, a subset of the original dataset was taken consisting of the 340 samples and merged with their corresponding CSF biomarker data. Given that the original ADNI diagnoses had been calculated purely on clinical measures, samples in this CSF subset were checked for accuracy of diagnosis according to amyloid positivity in CSF data as defined by a CSF amyloid value of below 880. This resulted in the removal of 14 AD samples and produced a final dataset of 326 samples and 146 proteins. Differential abundance analysis between AD and HC samples was performed with the R package Boruta (Kursa and Rudnicki, 2010), using a cut-off *p*-value of 0.05 and all other default parameters to obtain potential plasma biomarker proteins that were specific to a neuropathologically and clinically accurate AD diagnosis.

### 2.6 Differential single-cell transcriptomics analysis of brain tissue samples

The downloaded single-cell transcriptomics data of AD and healthy control entorhinal cortex samples consisted of the raw values of 10,850 genes in 13,214 cells which had been pre-annotated to microglia, neurons, endothelial cells, astrocytes, oligodendrocytes and oligodendrocyte precursor cells. Demographics are presented in Table 4. Subsequently, the dataset was subset into the six annotated cell types, and differentially expressed genes were obtained for endothelial cells, astrocytes, and microglia using the pipeline provided by the R package edgeR with TMM-wsp normalization (McCarthy et al., 2012; Robinson et al., 2010). The final adjusted cut-off *p*-value was set as 0.05.

**TABLE 4:** Demographics of single-cell transcriptomics data.

		AD	HC
n		6	6
Age^a^		80.8 (8.19)	79.2 (7.47)
Gender (Male/Female)		4/2	4/2
Number of cells	Total	5,764	6,120
	Neurons	249	407
	Microglia	172	277
	Oligodendrocytes	4,655	2,777
	Oligodendrocyte precursor cells	179	899
	Endothelial cells	37	61
	Astrocytes	472	1,699

^a^ Data is presented as mean (standard deviation).

### 2.7 Cell-cell communication analysis

Astrocyte-to-neuronal cell communication was assessed with the R package nichenetr (Browaeys et al., 2020). Briefly, astrocyte and neuron transcriptomics subsets were extracted and filtered for genes with less than 95% missing values. Expressed genes in astrocytes were then matched against ligands in a curated ligand-receptor database, and the extent to which expression of this gene was able to predict the downstream gene expression in neurons of a set of genes involved in neuronal death was calculated as a prediction score. Ligands expressed in astrocytes were ranked by prediction score, and interactions with relevant receptors on neurons were quantified.

### 2.8 Regulatory network analysis

Using the R package SCENIC (Aibar et al., 2017), gene regulatory networks were probed in astrocytes. Briefly, coexpression networks of transcription factors (TFs) with astrocytic genes were obtained using a called package GENIE3, which utilizes random forest models to determine the presence of relationships between TFs and other expressed genes. This process results in networks of coexpressed potential downstream genes helmed by a TF, known as a regulon. Regulatory motif enrichment on sequences surrounding the downstream gene start site is then performed, and genes without enriched motifs matching the binding site of its associated TF are pruned from the network. Finally, each cell is scored on regulon activity.

## 3 Results

### 3.1 Discovery of AD biomarkers upregulated in the AD plasma and brain proteome

40 proteins were significantly dysregulated (p-adj<0.05) in AD plasma samples compared to HC plasma samples, of which 29 were upregulated (Supplementary Table S1). In brain cortical tissue, a total of 1,111 proteins were found to be significantly dysregulated (p-adj<0.05) in AD *versus* HC samples, of which 499 were found to be upregulated (Supplementary Table S2). To obtain a putative set of BBM proteins transported from the brain, the intersection of the differentially upregulated proteins in both datasets was taken, resulting in a preliminary set of six proteins (Table 5). Haptoglobin was subsequently removed from the panel due to a lack of specificity to AD, resulting in a final panel of 5 BBM proteins, APOD, B2M, CFH, CLU and C3.

**TABLE 5:** Panel of BBM proteins.

Gene symbol	Protein	Fibrinogen correlation coefficient
APOD	Apolipoprotein D	-
B2M	Beta-2 microglobulin	0.240
CFH	Complement factor H	0.455
CLU	Clusterin	0.319
C3	Complement 3	0.657
HP	Haptoglobin	-^a^

^a^ Removed from further analyses due to nonspecificity to AD.

B2M, CFH, CLU and C3 were also found to be significantly correlated with brain fibrinogen levels after false-discovery correction (*p* < 0.05). This validates the presence of concurrent BBB dysregulation and BBM overabundance, lending support to the brain-to-blood leakage hypothesis.

### 3.2 Candidate BBMs are specific to AD

**FIGURE 3:**
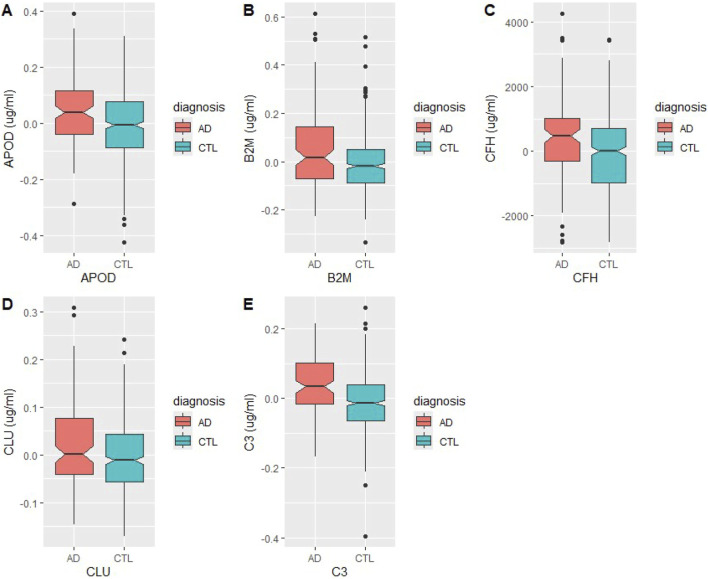
Levels of BBM proteins in Alzheimer’s Disease (AD) and healthy control (HC) plasma samples. Measurements were taken in µg/mL **(A)** Apolipoprotein D, **(B)** beta-2 microglobulin, **(C)** complement factor H, **(D)** clusterin, **(E)** complement 3.

To ascertain specificity of the BBMs to AD, association of the panel with alternative disease states was investigated. As mentioned, literature review of the candidate BBMs revealed that haptoglobin is a positive acute phase protein (Gulhar et al., 2023), a class of plasma proteins which is known to increase in response to general inflammation. As such, it was removed from further analyses as its increased abundance in AD samples was unlikely to be a result of brain-to-blood transport. The levels of each protein in AD and HC conditions in the final panel are shown in Figure 3. Using COVID-19 as a standard for systemic inflammation, the BBM panel was also validated to be independent of peripheral inflammation, based on published data of differentially abundant proteins in COVID-19 patient sera by (Shen et al., 2020).

To further rule out association of the BBM panel with generic neurodegenerative pathology, differential abundance analysis of plasma proteins was performed between plasma samples of HC with other neurodegenerative diseases, such as amyotrophic lateral sclerosis (ALS) and Parkinson’s disease (PD). None of the BBMs were dysregulated in the plasma samples of other neurodegenerative diseases (p-adj<0.05; Supplementary Table S3). Re-analysis of the original plasma proteomics data with CSF-corrected AD diagnoses also preserved APOD, CLU and C3 as specific BBMs of Aβ pathology. Results thus showed that the final panel of diagnostic BBMs, APOD, B2M, CFH, CLU and C3, were highly specific to AD diagnosis and its characteristic neuropathology, independently of noise from peripheral inflammation and neurodegeneration as a whole.

### 3.3 Predictive validity of the candidate BBM panel in a separate plasma proteomics dataset

**FIGURE 4:**
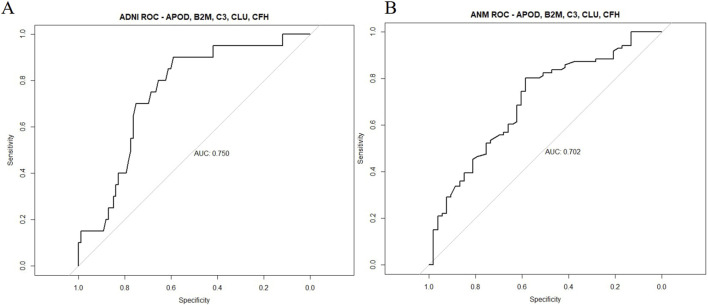
Predictive validity of the 5-protein BBM panel in the ADNI and ANMerge cohort. Random forest models were built with APOD, B2M, CFH, CLU and C3 levels as features for prediction of AD and HC. Datasets were separated with a 80-20 train-test split and assessed for predictive validity. **(A)** ADNI plasma proteome dataset **(B)** ANMerge plasma proteome dataset.

To assess predictive validity, ROC curves were built with random forest models trained on the original plasma dataset, as well as a novel ANMerge dataset, to investigate robustness of the BBM results. An AUC of 0.750 was achieved in the original dataset, and an AUC of 0.702 was obtained in the validation dataset (Figure 4). The BBM panel was therefore able to successfully differentiate AD from HC samples with a suitable level of accuracy and precision.

### 3.4 Upregulation of candidate BBMs is preserved at the mRNA level in AD astrocytes

Investigation into the mechanism of dysregulation of the BBMs in the brain with AD was performed after confirmation of the BBMs as valid and robust AD biomarkers. Since the hypothesis heavily implicated transportation through the blood-brain barrier, cell types in the neurovascular unit, namely, endothelia, astrocytes and microglia were probed with differential expression analysis. 215 genes were found to be differentially expressed in endothelial cells, while 2,450 genes were found to be differentially expressed in astrocytes and 337 were dysregulated in microglia. Differential expression of the BBM proteins was only observed in astrocytes, where *APOD*, *B2M*, *CLU* and *C3* were upregulated at the mRNA level in AD patients (Table 6). This provides evidence that the upregulated BBM proteins are brain derived.

**TABLE 6:** Log fold change of BBM proteins at the mRNA level in astrocytes.

Gene symbol	logFC^a^ in astrocytes
*APOD*	0.311
*B2M*	0.364
*CFH*	-
*CLU*	0.305
*C3*	0.968

^a^ log fold change.

### 3.5 BBM proteins are directly upregulated by AD-related gene regulatory processes and may be involved in neurodegeneration

Specificity of the BBMs to AD and evidence of transcript upregulation in AD astrocytes suggest that transcription and translation of the five proteins may be directly influenced by AD pathology. To investigate this, regulatory network analysis was performed on astrocytes. Further, a causative role of the BBMs in neuronal death was then investigated using cell-cell interaction analysis between neurons and astrocytes.

**FIGURE 5:**
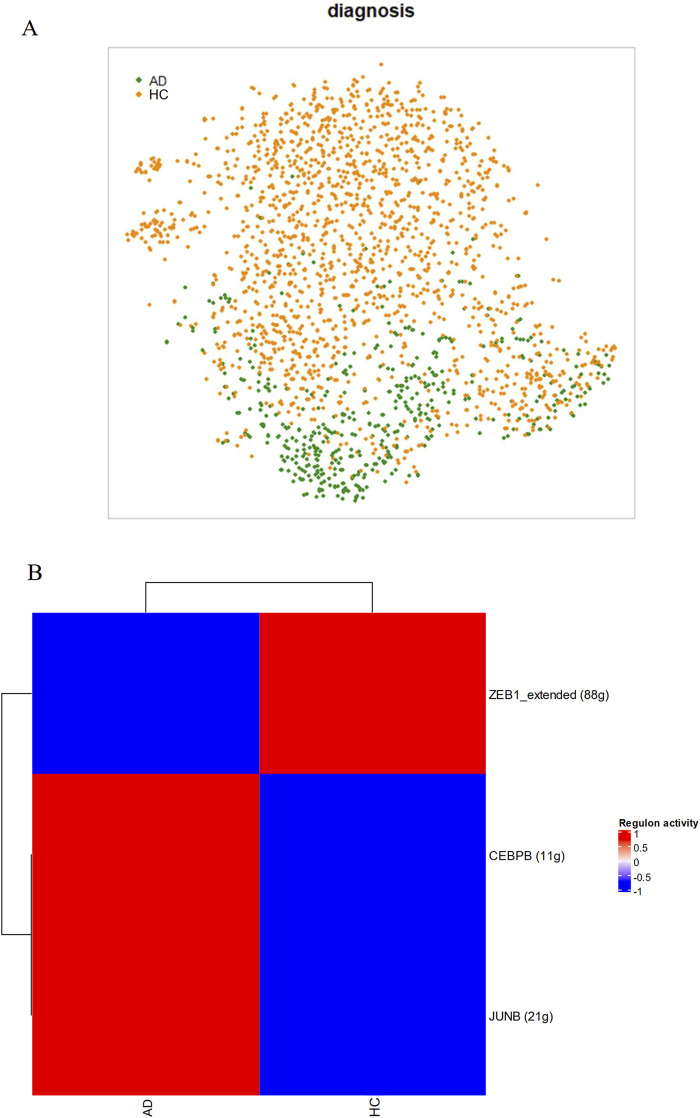
AD astrocytes have different regulatory network activities compared to HC astrocytes. As part of the SCENIC algorithm, single-cell transcriptomic data from astrocytes were subject to gene coexpression analyses with known TFs to generate regulatory networks. Membership was confirmed by presence of TF-binding motifs near the start site of the gene and cells were scored on regulon activity. **(A)** AD astrocytes segregated clearly in a t-SNE plot based on activities in 96 regulons and 50 principal components, **(B)** Scores generated by SCENIC on each regulon based on diagnosis.

Activity of the regulons identified by the SCENIC algorithm were generally able to distinguish between AD and HC astrocytes, forming two moderately segregated clusters with distinct centroids. This may suggest the presence of an astrocytic regulatory phenotype preferentially present in AD patients (Figure 5A). Figure 5B depicts the average direction of differential regulation of TFs in AD. Amongst the downregulated regulons, a disinhibition of *C3* was shown by the TF *ZEB1*. Of the TFs that showed increases in activities in AD, *CEBPB* and *JUNB* were identified as specific upstream regulators of *CLU* and *APOD* respectively.

**FIGURE 6:**
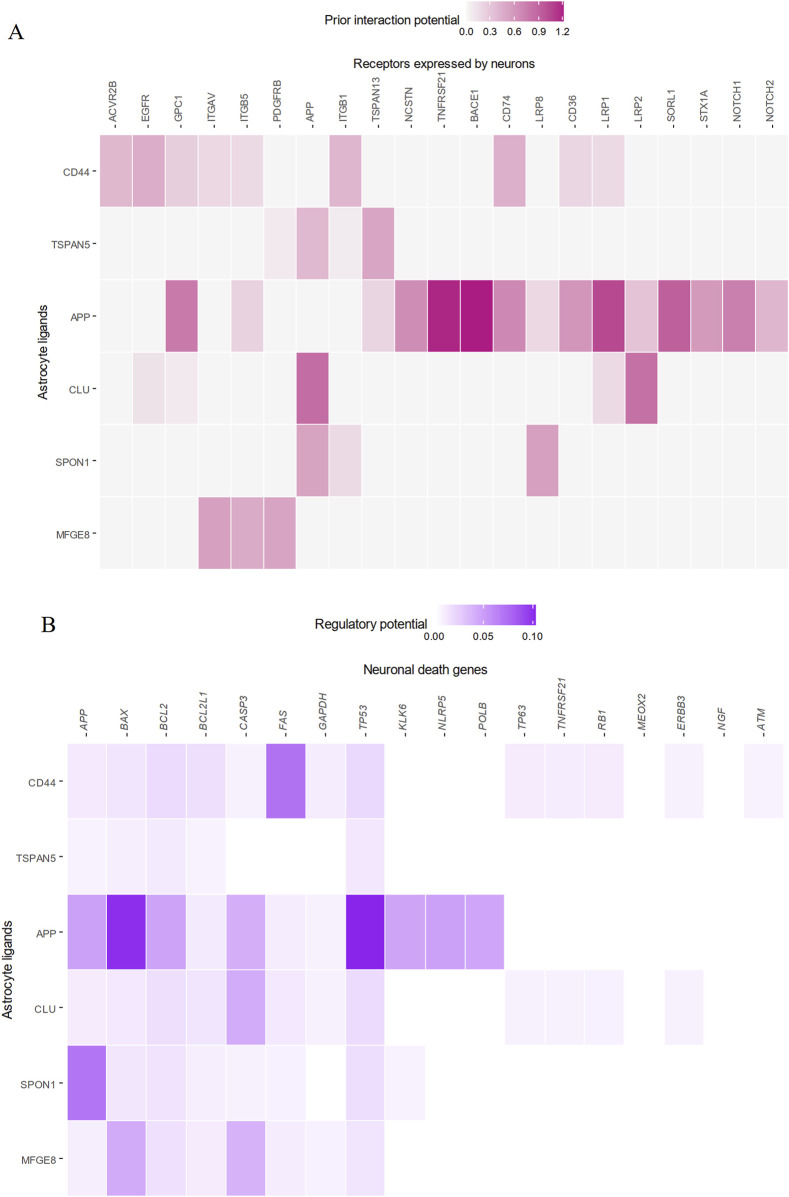
Astrocytic CLU is a significant regulator of neuronal apoptosis via interactions with LRP2 and APP. The top five astrocytic ligands best able to predict downstream neuronal death gene expression are plotted. **(A)** CLU from astrocytes was significantly co-expressed with LRP2 on neurons, and **(B)** was significantly able to predict downstream neuronal death genes.

Interestingly, cell-cell interaction analysis further revealed astrocytic CLU itself as a major upstream signaling regulator of genes involved in neuronal death (Figure 6A). *CLU* in astrocytes was co-expressed with the low-density lipoprotein-related protein two receptor (*LRP2*) and *APP* found on neurons (Figure 6B), and these proposed signaling interactions appeared to drive the upregulation of apoptosis genes *CASP3* and *BCL2* in neurons. Taken together, it can be concluded that increased activity of *CEBPB* in AD astrocytes enhances transcription and translation of CLU, and the increased amounts of CLU secreted interacts with neurons to directly trigger neuronal death and degeneration.

## 4 Discussion

Overall, this study identified five diagnostic BBMs that were able to differentiate AD samples from HC samples with AUCs of up to 0.75. These BBMs were upregulated in the plasma and brain proteome of AD patients, suggesting that the proteins may be brain-derived and may have leaked out into peripheral circulation as a result of increased BBB permeability seen in AD. The panel was not implicated in plasma protein changes during peripheral inflammation or in other neurodegenerative diseases such as PD or ALS, and was largely preserved in a neuropathology-corrected differential analysis, suggesting specificity to AD. Further supporting the source of the BBMs as the brain, transcripts of four proteins, APOD, B2M, CLU and C3 were found to be overexpressed in AD astrocytes. APOD, CLU and C3 were also highlighted as direct downstream targets of AD-dysregulated signaling pathways in astrocytes. In particular, it was also shown that upregulation of *CLU*, activated by the TF CEBPB, contributes to the neurodegenerative phenotype of the disease.

### 4.1 BBB permeability in AD

The core hypothesis of this study was that BBMs were upregulated in the AD brain and subsequently transported across the impaired BBB, of which a key assumption is the impairment of the BBB in AD. Transport across the BBB can occur by either paracellular or transcellular mechanisms, and both pathways are highly suppressed under healthy conditions (Sweeney et al., 2018). As such, damage to the BBB on top of brain proteomic dysregulation is required to transpose proteomic changes from the brain to the plasma (Das Gupta et al., 2019), thus suggesting a two-part mechanism for leakage of the BBMs into the plasma. In AD, BBB disruption encompasses a wide variety of phenotypes such as endothelial degeneration and pericyte loss (Sagare et al., 2013; Salmina et al., 2010; Yang et al., 2022a). Since only a limited number of proteins were found to be similarly upregulated in the brain and the plasma, a protein-specific mechanism that only allows certain proteins to cross the BBB into systemic circulation for detection as a BBM is necessary, thus favouring the receptor-mediated transcytosis (RMT) pathway over the nonspecific, size-dependent paracellular pathway.

Few studies investigate transcytosis across the BBB, especially with regard to AD. Nevertheless, some evidence does exist for the dysregulation of protein-specific transcytosis pathways in AD. Dysfunction of blood-to-brain transcytotic processes have been previously reported with age (Yang et al., 2022b), though protein transport in the reverse direction was not studied. As mentioned above, mechanisms of leakage of the five proteins through the BBB was unable to be identified in this study, however pathways for some have been proposed and experimentally validated in the literature. The BBMs and their hypothesised pathways are discussed below.

### 4.2 Significance of BBMs in AD

Robust evidence in the literature links the BBM panel to AD, with brain protein levels and upstream regulatory pathways tightly associated to either the induction or consequence of AD neuropathology. This provides a crucial evidence base substantiating why the five proteins may be specifically upregulated in the AD brain, and as such are able to be found in significantly larger amounts in the plasma of AD patients.

This study highlighted CLU as a downstream target of increased CEBPB activation with AD, which in turn enhances apoptotic signaling in neurons. Also known as ApoJ, the CLU protein is purported to perform many functions, including complement inhibition, the transport of lipids, as well as the induction of apoptosis [Foster]. Specifically in AD, CLU has been reported as a plasma biomarker for cognitive ability (Meng et al., 2015). In the brain, it co-localizes with Aβ plaques, and protects against AD by inhibiting Aβ aggregation (Chen et al., 2021; Spatharas et al., 2022; Wojtas et al., 2020), suggesting that upregulation of brain CLU in AD may be compensatory and triggered in response to increased Aβ load. Multiple single-nucleotide polymorphisms (SNPs) have been reported in the *CLU* gene which significantly increase risk of AD, suggested to be associated with dysregulated *CLU* transcript levels (Dauar et al., 2022; Szymanski et al., 2011). Most interestingly, CLU has also been ascribed a role in clearance of Aβ aggregates across the BBB, due to its interactions with LRP2 at the basal surface of endothelial BBB cells (Bell et al., 2007; Liu et al., 2022; Zlokovic et al., 1996), presenting an established and validated RMT pathway of CLU transport across the BBB. Interaction of astrocytic CLU with endothelial LRP2 was unable to be assessed in this study, as LRP2 mRNA was not reliably detected in endothelial cells, irrespective of diagnosis. This is supported by a well-documented difficulty in detecting LRP2 mRNA in cerebral microvessels, despite the fact that LRP2 proteins are consistently found in brain endothelia (Chun et al., 1999; Gosselet et al., 2009).

Another identified BBM of interest is the primary histocompatibility complex I subunit B2M. Primarily an immune response mediator [Li], upregulation of B2M in the AD brain may be a marker of the severity of AD pathology. B2M has been shown to exacerbate amyloid pathology in AD brains, and may be directly responsible for the neurotoxic effects of Aβ aggregation (Zhao et al., 2023). Increased plasma B2M levels have further been associated with cognitive performance (Huang et al., 2023). Cross-BBB transport of B2M has not yet been tied to any receptor (Smith et al., 2015), however transport across the proximal convoluted tubule in the kidney has interestingly been documented to be mediated by LRP2 (Argyropoulos et al., 2017; Nomura et al., 2014), the same receptor responsible for cross-BBB CLU transport. Further research into B2M transport across the BBB would be necessary to confirm such an association, however preliminary evidence may suggest dysregulation of LRP2 function on the AD BBB in relation to transcytotic protein leakage.

The remaining BBM proteins have received less attention in terms of identification of transcytotic transport mechanisms. However, upregulation of the proteins has been similarly implicated in AD pathology. APOD, a transporter of small hydrophobic molecules, has been shown to colocalize with Aβ plaques in the cortex (Muffat et al., 2008), and plays a protective role by decreasing the expression of the β-amyloid precursor protein in astroglial cell cultures (Bhatia et al., 2019; Dassati et al., 2014; Rassart et al., 2020). Upregulation of APOD may thereby aid in reducing plaque load (Li et al., 2015). C3, one of the most robustly replicated plasma BBMs of AD, is a crucial modulator of Aβ plaque-associated synaptic loss via interactions between astrocytes, the major producer of C3, and microglia (Batista et al., 2024; Hong et al., 2016; Shi et al., 2017; Wu et al., 2019). Finally, the complement pathway regulator CFH has been identified as a genetic risk factor for AD, where risk alleles represent dysregulations in *CFH* transcript levels (Veteleanu et al., 2023). AD-related *CFH* SNPs are shown to affect cortical thickness of the entorhinal cortex in AD patients, suggesting a CFH-driven increase in cortical atrophy rate (Zhang et al., 2016).

Upstream of the increased levels of candidate proteins in the brain, AD-related dysregulation of astrocytic transcription signalling pathways was also revealed, namely, upregulation of CEBPB and JUNB pathways and downregulation of the ZEB1 pathway. Activity of the CCAAT-enhancer-binding TF CEBPB in glia cells has been previously shown to be directly enhanced by the presence of Aβ (Ramberg et al., 2011; Yao et al., 1833-1852), suggesting a direct link between AD pathology and CLU upregulation. Other downstream effects of CEBPB signaling include cytokine production, which causes neuroinflammation, as well as upregulation of δ-secretase, a key mediator of AD onset and progression which acts by cleaving both tau and Aβ to trigger formation of plaques and neurofibrillary tangles (Wang et al., 2018). However, while CEBPB signaling is strongly associated with AD in literature, ZEB1 and JUNB pathways, which regulate C3 and APOD respectively, are less well characterized. Outside of AD, the ZEB1 regulatory network has been implicated in neuroinflammation and astrogliosis (Poonaki et al., 2022), and interestingly, can regulate permeability of BBB endothelia under hypoxia (Leduc-Galindo et al., 2019). Additionally, while the JUNB regulatory network has not been implicated in AD processes, the kinase signaling pathway of its TF binding partner, cJun, has been consistently proven as a regulator of many AD-related phenotypes, such as cognitive impairment, altered synaptic function, glial clearance of Aβ plaques and neuronal death (MacDonald et al., 2013; Sclip et al., 2014; Scopa et al., 2023). Investigation of these novel signaling pathways in AD glial cells may be useful to further our understanding of the molecular complexity of AD effects on brain pathology.

### 4.3 Limitations and future work

Most primarily, this study is limited by a lack of direct evidence supporting the increased BBB leakage mechanism of the BBMs into AD systemic circulation. This is as BBB changes are difficult to observe in proteomic or transcriptomic data, and largely rely on neuroimaging data or post-mortem brain tissue analysis for detection (Barisano et al., 2022). Thus, as a purely data study, validation of increased protein transport across the BBB was unable to be performed, potentially undermining the biological relevance of 5 BBMs. In addition, AD has been increasingly recognized as a stagewise, progressive disease, beginning from a healthy stage to one of subjective memory complaints, mild cognitive impairment (prodromal AD), and finally full-blown AD (Kim et al., 2022). As such, investigating changes in the plasma proteome in a stagewise manner and over a longitudinal time frame may be better suited for management of this disease to identify individuals at risk of progressing along the trajectory (Guo et al., 2024), rather than the cross-sectional methodology applied here.

Nevertheless, the integration of multiple datasets across three different data modalities employed in this study provides a measure of reliability and increases confidence in the validity of the identified BBMs, allowing the panel to serve as a preliminary theoretical foundation on which future investigations can be based on. Robust specificity analyses eliminating noise from peripheral inflammation and general neurodegenerative processes further substantiate the panel’s efficacy in AD diagnosis. Although the AUROCs obtained in this study may not reach standards achieved by previously published BBM panels (Jiang et al., 2022; Wang et al., 2023), its theory-based approach may lend itself to greater replicability in different samples and populations, especially crucial in a field where inter-study heterogeneity is a significant problem. Undoubtedly, future studies embarking on clinical validation of the protein panel in a real-world population of AD and HC samples are necessary to conclude on the efficacy of the panel as AD BBMs.

To conclude, 5 BBMs specifically dysregulated in AD were obtained for AD diagnosis. Panel proteins were shown to be upregulated in the brain in response to AD brain neuropathology, supporting the brain source hypothesis, and may leak out into the blood through dysregulated LRP family receptors on disrupted BBB endothelia which mediate transcytosis across the BBB. Discovery of this panel has implications on future clinical use, and may also serve as a foundation for further research into brain-to-blood protein transport in diseases involving BBB disruption.

## Data Availability

The original contributions presented in the study are included in the article/Supplementary Material, further inquiries can be directed to the corresponding author. The datasets analysed for this study can be found on the AD Knowledge Portal (https://adknowledgeportal.org), the ADNI online database (http://adni.loni.usc.edu).
